# Successful Treatment of Acute Bilateral Pulmonary Thromboembolism Using Accelerated Regimen of Thrombolytics: A Case Report

**DOI:** 10.7759/cureus.87600

**Published:** 2025-07-09

**Authors:** Thanigasalam Tharsiga, Gamini Galappatthy, Disna Amaratunga

**Affiliations:** 1 Internal Medicine, National Hospital of Sri Lanka, Colombo, LKA; 2 Cardiology Unit, National Hospital of Sri Lanka, Colombo, LKA

**Keywords:** accelerated regimen, acute pulmonary thromboembolism, pulmonary embolism severity index, recombinant tissue plasminogen activator, right ventricular dysfunction

## Abstract

Acute pulmonary thromboembolism represents a critical, life-threatening condition that necessitates immediate and effective management to minimize associated mortality. Although the current recommendation for managing hemodynamically unstable pulmonary embolism (PE) is the regimen of 100 mg recombinant tissue plasminogen activator (rtPA) administered over two hours, an accelerated regimen of rtPA (0.6 mg/kg over 15 minutes, maximum 50 mg) has also been described for the management of PE and circulatory arrest. We report the case of a 65-year-old female patient who presented with acute bilateral pulmonary thromboembolism at intermediate-high risk. She was treated with an accelerated rtPA regimen, following which she demonstrated significant clinical improvement. Her oxygen saturation improved from 88% to 96%, and her respiratory rate decreased from 36 to 20 breaths per minute. Hemodynamic parameters remained stable throughout treatment. A computed tomography pulmonary angiogram (CTPA) performed on day 4 showed marked resolution of thrombi and improved pulmonary perfusion. No major bleeding complications occurred, and the patient was safely transitioned to oral anticoagulation. This case underscores the potential noninferiority of the accelerated thrombolysis regimen compared to the conventional two-hour regimen in terms of mortality and pulmonary vascular resistance, with a tendency toward fewer bleeding complications observed with the accelerated regimen.

## Introduction

Pulmonary embolism (PE) ranks as the third most common cause of death, following ST-elevation myocardial infarction and stroke [1,2]. It is also a major contributor to mortality in certain high-risk populations, such as pregnant women, cancer patients, individuals with traumatic injuries, and the very elderly. Recent findings show that in very elderly patients with PE, thrombolysis is often considered based on signs of imminent clinical decline, not just low blood pressure [1,2].

While thrombolytic therapy has been shown to enhance outcomes in cases of high-risk and intermediate-high risk PE, its use remains debated due to the elevated risk of intracranial hemorrhage, particularly in patients over the age of 60 [3]. Although the current guidelines recommend the regimen of 100 mg recombinant tissue plasminogen activator (rtPA) over two hours to be used for hemodynamic instable PE, an accelerated regimen of rtPA (0.6 mg/kg over 15 minutes, maximum 50 mg) has been mentioned for the management of PE and circulatory arrest in the 2019 European Society of Cardiology and the European Respiratory Society (ESC/ERS) guidelines. Nevertheless, no empirical evidence is provided to validate this assertion. Here, we present a case of acute bilateral PE successfully managed using an accelerated regimen of rtPA.

## Case presentation

A 65-year-old female patient presented to the cardiology department with worsening shortness of breath associated with nonischemic chest discomfort for one week and left lower limb swelling for two weeks. Her past medical history was significant for cervical cancer diagnosed two years ago, for which she was receiving ayurvedic treatment. She also had a history of varicose veins and left lower limb deep vein thrombosis 15 years ago, for which she was on warfarin but defaulted on treatment. Additionally, she had hypertension for the past five years and was on antihypertensive medications.

On examination, she was obese with a body mass index of 36.4 kg/m^2^. She was dyspneic, with an SpO2 of 88% on room air and a respiratory rate of 36 breaths/minute. Auscultation revealed vesicular breath sounds without added sounds. Her blood pressure was 120/80 mmHg, and her pulse rate was 98 bpm. A pansystolic murmur was best heard at the left lower sternal edge without radiating to the axilla, suggestive of tricuspid regurgitation, along with a loud P2. The left lower limb showed erythema and nontender edema.

**Figure 1:**
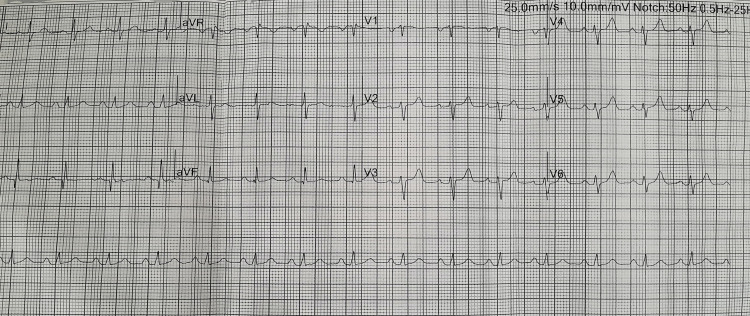
Electrocardiogram showing sinus tachycardia and an S1Q3T3 pattern

**Figure 2:**
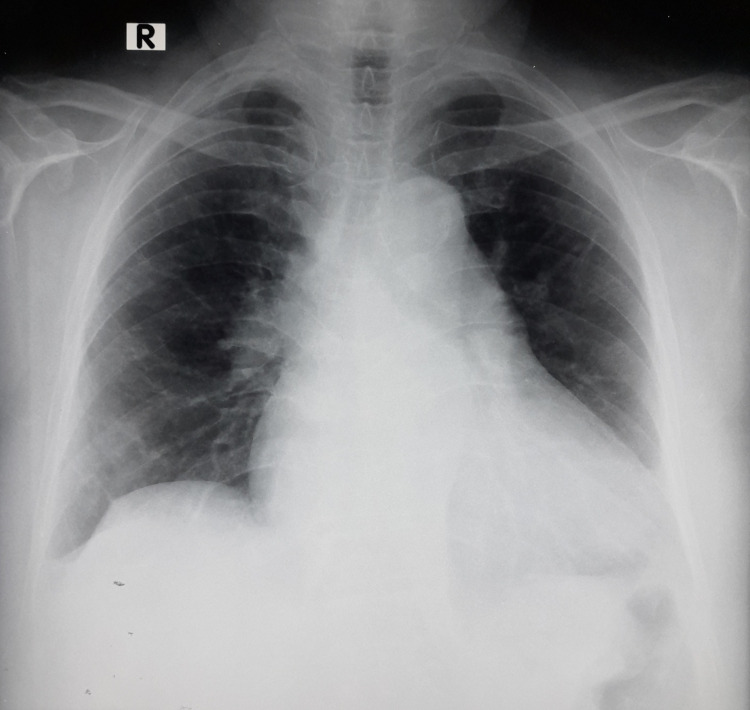
Chest X-ray (PA) demonstrating cardiomegaly PA: posteroanterior

**Figure 3:**
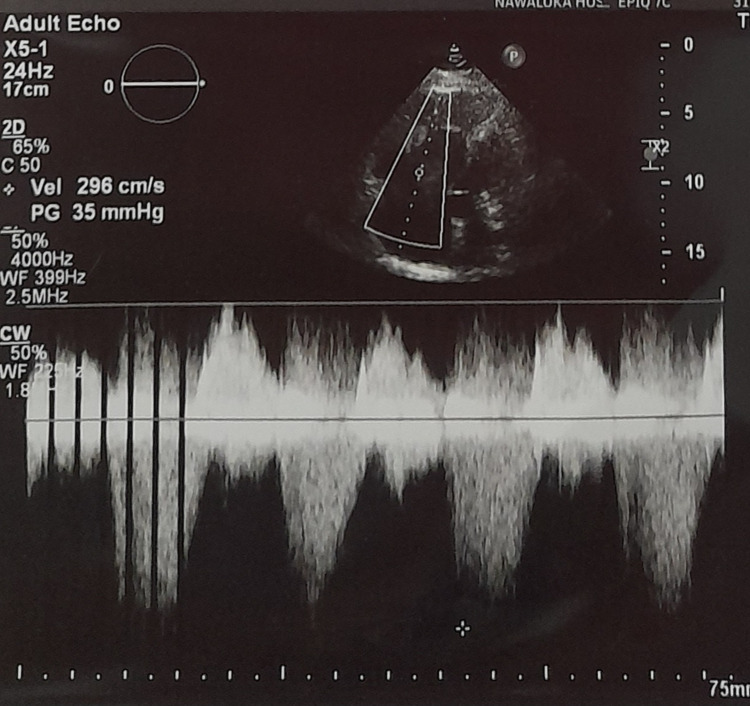
Transthoracic echocardiogram demonstrating a grossly dilated right atrium and right ventricle

Table 1 summarizes the laboratory and biomarker findings. High-sensitivity troponin level was elevated. The electrocardiogram showed sinus tachycardia and an S1Q3T3 pattern (Figure 1). Chest X-ray revealed cardiomegaly (Figure 2). Transthoracic echocardiogram demonstrated a grossly dilated right atrium and right ventricle, a dilated pulmonary artery, severe pulmonary hypertension, and right ventricular (RV) dysfunction with a tricuspid annular plane systolic excursion (TAPSE) of 14 mm (Figure 3). An initial diagnosis of acute intermediate-high risk pulmonary thromboembolism was considered, supported by a high-probability Wells score assessment.

**Table 1:** Summary of investigations WBC: white blood cell; RDW-CV: red blood cell distribution width-coefficient of variation; eGFR: estimated glomerular filtration rate; INR: international normalized ratio

Test (unit)	Value	Reference range
High sensitivity troponin I (ng/L)	38	>16
WBC (×10^3^/uL)	9.01	4.0-11.0
Hemoglobin (g/dL)	11.8	12.0-16.0
Mean corpuscular volume (fL)	88.9	80.0-100.0
Mean corpuscular hemoglobin concentration (g/dL)	28.1	32.0-36.0
RDW-CV (%)	14.6	11.0-16.0
Platelet (×10^3^/uL)	240	150-450
Serum sodium (mmol/L)	136	136-145
Serum potassium (mmol/L)	4.8	3.5-5.1
Aspartate transaminase (U/L)	58	15-37
Alanine transaminase (U/L)	65	12-78
Serum creatinine (mg/dL)	0.85	0.55-1.02
eGFR (ml/min/1.73m^2^)	78	58-130
INR	1.46	1

**Figure 4:**
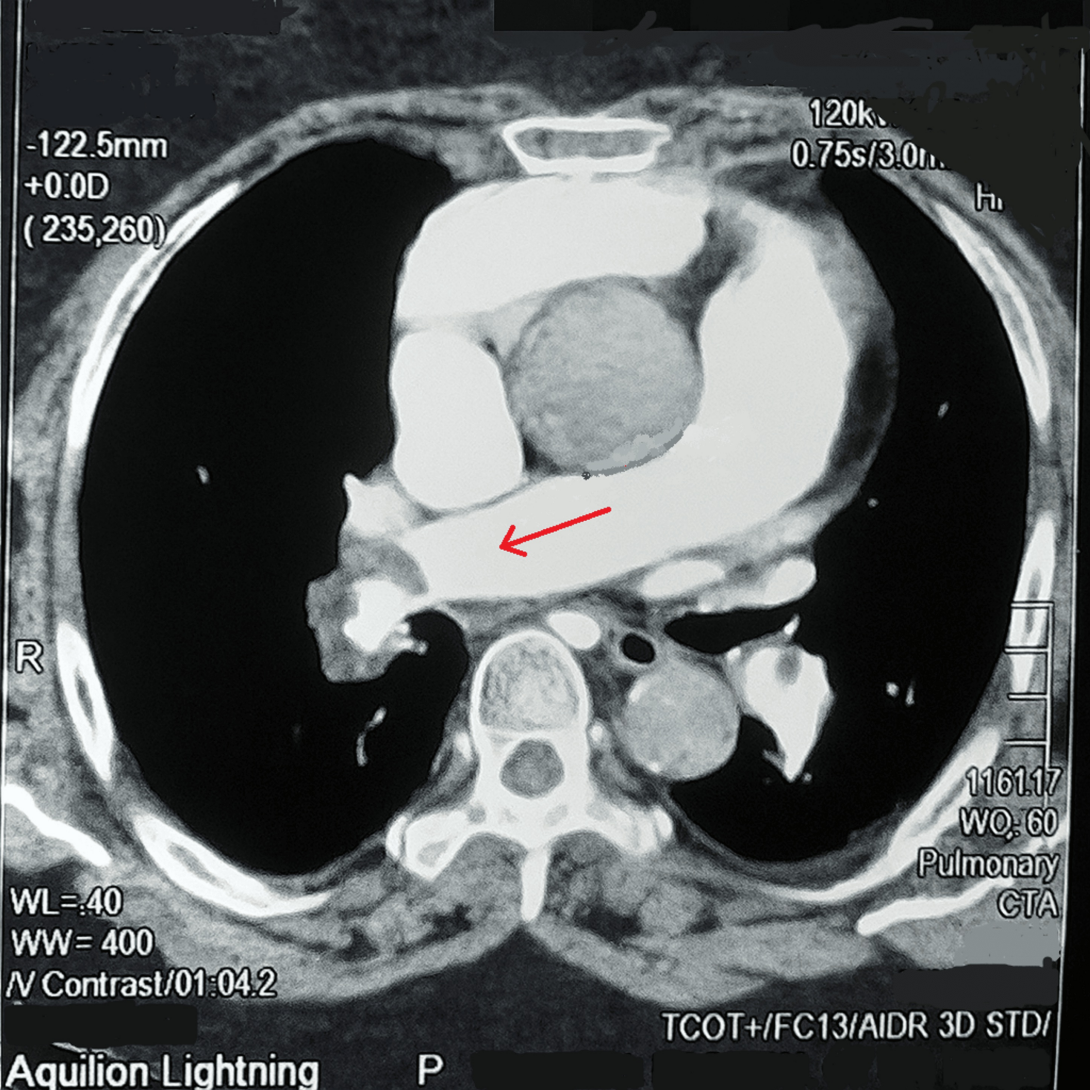
Computed tomography pulmonary angiogram (CTPA) demonstrating a filling defect in the right main pulmonary artery, partially occluding the vessel

Initial computed tomography pulmonary angiogram (CTPA) demonstrated a filling defect in the right main pulmonary artery, partially occluding the vessel (Figure 4). The filling defect extended into the segmental arteries supplying all three lobes. A small wedge-shaped area was seen in the superior segment of the right lower lobe, and another similar but smaller area in the apical segment of the right upper lobe, likely representing small areas of pulmonary infarction. Filling defects were also noted in the segmental arteries of the left upper lobe, while the arteries supplying the lower lobe were spared. Based on these findings, a diagnosis of acute bilateral pulmonary thromboembolism was confirmed, and intravenous thrombolysis with rTPA was initiated.

The patient was managed in the intensive care unit. It was planned to administer a modified thrombolytic regimen. Intravenous alteplase 50 mg (0.6 mg/kg) was administered over 15 minutes. Intravenous heparin infusion was withheld as the patient was experiencing pervaginal bleeding. Subcutaneous enoxaparin 80 mg twice daily was initiated once the bleeding had resolved. No other bleeding manifestations were observed. The following day, her oxygen requirement decreased, and there was moderate improvement in her symptoms. On day 4 of thrombolytic therapy, a repeat CTPA was performed, which showed improvement in the filling defects compared to the previous scan.

As there was no further bleeding, she was started on oral warfarin therapy while continuing enoxaparin. Her clinical status continued to improve over the next two days, and as she no longer required oxygen therapy, she was transferred to the ward for further observation and INR optimization. During her ward stay, she remained asymptomatic, and the following day, she was discharged with oral warfarin. A gynecological referral was made for ongoing management of her cervical cancer.

## Discussion

Pulmonary thromboembolism occurs when thrombi, typically originating from the deep veins, migrate and obstruct the pulmonary arteries. This condition poses a serious threat to life by compromising both blood circulation and oxygenation, and in severe cases, may result in RV failure [4]. Risk factors contributing to the development of venous thrombi include major surgical procedures, significant trauma, hormone replacement therapy, advanced age (over 40 years), malignancy, prolonged immobility exceeding three days, and a prior history of thromboembolic events. These thrombi can dislodge and embolize, leading to blockage of the pulmonary vasculature [4].

The initial assessment of mortality risk of PE is done using the PE severity index (PESI) or simplified PESI (sPESI) (Table 2) and evaluation of the RV function through imaging computerized tomography and/or echocardiogram, in conjunction with biochemical markers including troponin, brain natriuretic peptide (BNP), and N-terminal pro-BNP [5]. Low risk PE is characterized by hemodynamic stability, normal RV size and function, and unremarkable RV biomarkers, and a PESI class І or ІІ (score<86) or a sPESI score of zero. Intermediate-risk PE, also referred to as submassive PE, is defined by the presence of RV dysfunction in the absence of systemic hypotension. This category typically corresponds to a PESI class ІІІ to Ⅴ (score>=86) or a sPESI score of 1 or higher. It is further subdivided into intermediate-low risk (abnormal RV function or elevated biomarkers) and intermediate-high risk (abnormal RV function and elevated biomarkers). These subgroups within the intermediate-risk category assist in stratifying patients based on mortality risk and identifying those who may derive clinical benefit from thrombolytic therapy. High-risk PE, also referred to as massive or unstable PE, encompasses a heterogeneous group of patients, ranging from those presenting with systemic hypotension to individuals experiencing cardiac arrest or obstructive shock [6]. In our case, the patient had several risk factors, including cervical cancer, advanced age, and a history of deep vein thrombosis. She presented with dyspnea and chest discomfort, along with RV dysfunction evidenced by imaging, elevated troponin I level, and a PESI class Ⅴ (score of 135) or sPESI score of 2. She was categorized as intermediate-high risk, where thrombolytic therapy may be beneficial.

**Table 2:** Pulmonary embolism severity index scores: full and simplified

Pulmonary embolism severity index (PESI)-full		
Clinical features		Points
Age		X (e.g., 65)
Male gender		10
History of cancer		30
Heart failure		10
Chronic lung disease		10
Pulse >= 110/min		20
Systolic blood pressure < 100mmHg		30
Respiratory rate >= 30min		20
Temperature <36℃		20
Altered mental status		60
Arterial oxygen saturation <90%		20
Class Ⅰ	Low risk	<66
Class Ⅱ		66 to 85
Class Ⅲ		86 to 105
Class Ⅳ	High risk	106 to 125
Class Ⅴ		>125
Simplified pulmonary embolism severity index (sPESI)		
Age >80 years		1
History of cancer		1
Chronic cardiopulmonary disease		1
Pulse >= 110/min		1
Systolic blood pressure < 100mmHg		1
Arterial oxygen saturation <90%		1
Low risk		0
High risk		>= 1

Thrombolytic therapy has demonstrated greater efficacy in thrombus dissolution compared to the combined use of heparin and warfarin. Beyond its clot-resolving properties, early therapeutic advantages include improved RV function and preservation of pulmonary diffusing capacity [7]. The existing literature is more extensive for patients with massive or submassive PE who do not present with circulatory arrest. Notably, the two largest randomized controlled trials evaluated an accelerated thrombolytic regimen (0.6 mg/kg/15 min, up to a maximum of 50 mg) against the standard two-hour 100 mg regimen. Both trials employed nearly identical methodologies and were published concurrently. Individually, neither study demonstrated significant differences in outcomes related to mortality, pulmonary vascular resistance, perfusion defects, or bleeding complications [8,9].

A meta-analysis encompassing both trials reported no significant difference in overall clinical outcomes; however, it identified a trend toward reduced bleeding rates with the accelerated thrombolytic regimen (11% vs 20%, p = 0.19) [10]. This potential reduction in bleeding complications may be attributed to the dose-dependent impact on fibrinogen levels and related biomarkers. A subanalysis from one of the studies demonstrated significantly lower reductions in fibrinogen, fibrin degradation products, and plasmin-α2-antiplasmin complexes in the accelerated regimen [11].

A more recent meta-analysis incorporated an additional randomized controlled trial that evaluated a reduced-dose, nonaccelerated thrombolytic regimen (rtPA 50 mg over two hours) compared to the standard 100 mg over two hours [12]. This analysis demonstrated a statistically significant reduction in bleeding events (odds ratio: 0.33; 95% CI: 0.12-0.91), while no significant difference in mortality was observed (odds ratio: 0.88; 95% CI: 0.23-3.37) [13]. Our patient was given an accelerated regimen of rtPA (alteplase) and showed significant improvement in symptoms and perfusion defects, supporting the abovementioned evidence.

## Conclusions

Acute pulmonary thromboembolism is a frequently under-recognized clinical entity that can be fatal if not promptly treated. Early diagnosis and timely initiation of thrombolytic therapy are critical for significantly reducing associated mortality. The conventional systemic thrombolytic regimen involves administering intravenous alteplase at 100 mg over two hours. However, successful thrombolysis can also be achieved with an accelerated regimen (0.6 mg/kg over 15 minutes, maximum dose 50 mg), which is associated with a lower risk of bleeding.
